# Role of T Cells in Microbial Pathogenesis

**DOI:** 10.3390/pathogens12111321

**Published:** 2023-11-06

**Authors:** Liqing Wang, Jianxun Song

**Affiliations:** 1 Department of Microbial Pathogenesis and Immunology, Texas A&M University Health Science Center, Bryan, TX 77807, USA; wang1989@tamu.edu; 2 Department of Biochemistry and Biophysics, Texas A&M University, College Station, TX 77843, USA

The immune system functions as a sophisticated defense mechanism, shielding the body from harmful pathogenic invaders. Various types of immune cells collaborate in response to pathogenic infections, with T cells emerging as prominent frontline warriors within the adaptive immune response. In this outline, we will succinctly explore T cell responses to pathogenic infections, emphasizing their pivotal role in counteracting invaders and upholding immune memory.

Upon the intrusion of a pathogenic infection, specialized antigen-presenting cells (APCs) identify and engulf the trespassers, subsequently breaking them down into smaller fragments. These pathogen fragments are then presented by APCs to activate naive T cells. Once triggered, T cells rapidly undergo proliferation and differentiation into effector T cells. Among them, CD8^+^ T cells assume the role of frontline assassins, employing perforin and granzymes to eliminate infected cells. Concurrently, CD4^+^ T cells orchestrate the immune response by aiding B cells in antibody production and bolstering macrophages in their defense against invaders.

Upon the successful clearance of the infecting pathogens, a subset of cells persists, referred to as memory T cells. These memory T cells strategically position themselves throughout the body, poised to mount a swifter and more robust response upon encountering the same pathogen again. This phenomenon significantly heightens protection against potential reinfection.

Previous research has delved into T cell responses to a diverse array of pathogens, encompassing viruses, bacteria, fungi, and parasites. Notably, researchers have dedicated substantial efforts to scrutinizing the impact of T cell responses on SARS-CoV-2, as evidenced by a multitude of studies [1,2]. In the context of *Mycobacterium tuberculosis* infection or co-infection scenarios involving other viral agents, both CD8^+^ and CD4^+^ T cells play pivotal roles in orchestrating responses [3]. Focusing on fungal recognition and the reinforcement of antifungal immunity within T helper cells, C-type lectin receptors emerge as indispensable factors [4]. The utilization of *Toxoplasma gondii* as a trigger for successful CD8^+^ responses in mice underscores the significance of such responses in combatting this particular pathogen [5].

Simultaneously, various factors can disrupt T cell responses during pathogenic infections. These factors encompass diverse elements such as pathogen types, cytokine production, T cell transcriptional factors, and co-stimulatory factors, all of which play intricate roles in influencing and disrupting T cell immune responses. An illustrative example is the impact of HIV on the host’s immune system, achieved by depleting CD4^+^ helper T cells and consequently undermining immune defenses [6]. Similarly, chronic HBV infection tends to induce latency and trigger only limited T cell responses [7]. Essential to T cell activation is IL-1, while IL-2 assumes a pivotal role in fostering T cell proliferation [8]. In the fight against pathogenic infections, T cells secrete cytokines such as IFN-γ, Granzyme B, and perforin, contributing to defense mechanisms [9,10]. The transcriptional factor FoxO1 has been identified as a promoter of antiviral CD8^+^ T cell survival in the context of chronic infections [11].

Alterations in T cell metabolism play a pivotal role in determining the performance of T cells during pathogenic infections. As T cells transition from naive to effector and memory states, their metabolic requirements shift accordingly. These metabolic activities encompass glycolysis, oxidative phosphorylation (OXPHOS), and fatty acid oxidation (FAO) [12]. An illustrative case is the examination of T cells derived from individuals afflicted with SARS-CoV-2. In these patients, T cells exhibit discernible mitochondrial metabolic deficiencies [13]. Similarly, in the context of chronic infections, researchers have observed changes in T cell metabolism among exhausted T cells, further underscoring the intricate connection between metabolic status and T cell function [14].

Within the realm of pathogenic infections, a crucial facet concerning T cells is the phenomenon of exhaustion. This occurrence gives rise to dysfunctional T cells, manifesting in various scenarios including chronic viral infections or within the context of a cancerous environment [15]. Regrettably, these exhausted T cells fail to operate optimally, resulting in a diminished capacity to combat viruses or tumor cells. An apt illustration can be found in the interaction between CD4^+^ and CD8^+^ T cells during *M. tuberculosis* infection. CD4^+^ T cells play a role in supporting CD8^+^ T cells and thwarting their exhaustion [16]. In parallel, chronic LCMV infection leads to the exhaustion of CD8^+^ T cells and dampens TCR signaling [17]. Furthermore, individuals carrying HBV commonly exhibit a prevalence of exhausted circulating CD8^+^ T cells [18].

Subsequent to a host’s recovery from a pathogenic infection, the development of memory T cells ensues. The formation of these memory T cells is subject to the influence of multiple factors, encompassing co-stimulatory and transcriptional elements. Notably, co-stimulatory factors, in conjunction with transcriptional factors, orchestrate the intricate process of T cell memory formation. A pertinent example of a co-stimulatory factor’s significance is CD28, which assumes a critical role in facilitating the creation of memory T cells [19]. Similarly, the role of 4-1BB cannot be overstated in the establishment of virus-specific tissue-resident memory CD8^+^ T cells, particularly within the lung environment [20]. Transcriptional factors also play a defining role, as evidenced by NAC1’s regulatory impact. Research has indicated that NAC1 functions as a limiter for both CD4^+^ and CD8^+^ memory T cell formation during viral infections [21,22]. These intricate interactions between co-stimulatory and transcriptional factors contribute to the finely tuned process of T cell memory formation.

T cell responses to pathogenic infections vividly illustrate the remarkable efficiency and precision of the adaptive immune system. Through intricate interplays, T cells orchestrate the elimination of invaders and the establishment of immune memory. Investigating the roles played by T cells in the context of pathogenic infections holds profound significance, offering insights crucial for the development of potent vaccines and therapies aimed at combating infectious diseases and fostering overall well-being. The enhancement of T cell functionality, optimization of T cell-mediated cytokine responses, extension of T cell memory duration, and prevention of T cell exhaustion emerge as potential strategies to bolster the host’s immune system. These strategies not only hold promise for enhancing T cell performance, but also offer avenues for T cell-based immunotherapy. Notably, these strategies can find applications not only in the realm of pathogenic infections, but also in the realm of cancer treatment, showcasing the broad spectrum of their potential impact.
